# Using Stream Data Processing for Real-Time Occupancy Detection in Smart Buildings

**DOI:** 10.3390/s22062371

**Published:** 2022-03-19

**Authors:** Hamza Elkhoukhi, Mohamed Bakhouya, Driss El Ouadghiri, Majdoulayne Hanifi

**Affiliations:** 1 LERMA-Lab, College of Engineering and Architecture, International University of Rabat, Sala El Jadida 11103, Morocco; mohamed.bakhouya@uir.ac.ma (M.B.); majdoulayne.hanifi@uir.ac.ma (M.H.); 2 IA Lab, Science Faculty, My Ismail University, Meknès 11201, Morocco; d.elouadghiri@umi.ac.ma

**Keywords:** occupancy detection, internet of things, energy efficiency in buildings, streaming machine learning, stream data processing

## Abstract

Controlling active and passive systems in buildings with the aim of optimizing energy efficiency and maintaining occupants’ comfort is the major task of building management systems. However, most of these systems use a predefined configuration, which usually do not match the occupants’ preferences. Therefore, occupancy detection is imperative for energy use management mainly in residential and industrial buildings. Most works related to data-driven-based occupancy detection have used batch learning techniques, which need to store first and then train the data. It is not appropriate for a non-stationary environment. Therefore, this work sheds more light on the use of non-stationary machine learning techniques. To this end, three machine learning algorithms for stream data processing are presented, tested, and evaluated in term of accuracy and resources performance (i.e., RAM, CPU), with the aim of predicting the number of occupants in smart buildings. A platform architecture that integrates IoT technologies with stream machine learning is implemented and deployed. The experimental results show the effectiveness of this approach and illustrate that the number of occupants can be predicted with an accuracy of more than 83% and without resource wasting (i.e., time of CPU use varied between 0.04s and 3.85 ⋅ 10^−11^ GB of RAM could be exploited per hour).

## 1. Introduction and Motivation

According to the Moroccan Agency for Energy Efficiency (AMEE), the Moroccan industrial sector represents 21% of the total energy consumption. In fact, Morocco imports more than 90% of its energy needs. Rising energy needs are expected due to its population growth and industrial development [1]. To face these challenges, the government has developed a new strategy to secure energy supplies while protecting the environment. The Moroccan strategy mainly aims to improve the energy efficiency approaches in the industrial sector. The obligation of energy audits in this sector is an important plan for the Moroccan government to achieve the targeted ratio of energy savings of 20% by 2030, as stated in the 12th Article of the 47-09 law, which is relative to energy efficiency [2]. According to the Moroccan plan for energy efficiency in buildings, several research works have shed more light on the development of building management systems (BMSs), with the goal of minimizing energy consumption and maintaining the occupants’ comfort. In fact, the goal of BMSs in commercial structures is to provide optimal conditions regarding indoor air quality (CO_2_ and humidity), thermal parameters, and lighting of workplaces and common areas (illuminance).

Likewise, many solutions of BMSs aim to control access to protected areas in buildings, which requires occupancy monitoring [3]. Furthermore, 2020’s pandemic (COVID-19) precaution rules also call for integrating occupancy estimation methods in commercial buildings, and balancing the supply air and exhaust air between HVAC air handling units and local extraction fans to ensure good air quality in shared spaces. In fact, the efficiency of the BMS is directly linked to the tradeoff between the quantity of energy consumed and the occupants’ comfort in buildings. Several research works have investigated the development of methods, which aim to control HVACs and lighting systems based on occupancy estimation. Recently, several studies related to the energy efficiency of buildings have focused on more particular types of occupant interactions and their impact on energy consumption [4]. Most stated that a building’s elements in control systems can be operated according to the occupancy, aiming to achieve a desired level of comfort in different ways, such as building openings (e.g., opening and closing windows), HVAC systems control (e.g., turning air-conditioning on or off and adjusting the thermostat temperature), lighting and shading (e.g., adjusting blinds), and electrical appliances [5]. For instance, HVAC-L systems are the main elements that allow occupants to achieve their own thermal and visual comfort, which are the key sources of energy consumption in buildings. Therefore, indoor occupancy is considered the major factor that influences residential and commercial buildings’ overall energy consumption. In fact, several studies have investigated the development of solutions that correlate energy consumption to the activities of a building’s occupants’. For example, the work presented in [6,7] proposes a non-intrusive occupant load monitoring approach (NIOLM) that links occupancy information using already deployed Wi-Fi infrastructure for power variation in a building by tracking 11 occupants’ energy exploitation. Additionally, comprehensive and ground truth occupancy information is considered as the main input to control the building’s active systems. Furthermore, it could be categorized into different parameters and properties. In fact, recent research studies related to occupancy detection approaches presented six spatial-temporal properties (i.e., presence, count, location, activity, identity, track) in order of importance regarding the correlation with building energy consumption [8,9]. However, to infer these types of parameters, it is necessary to exploit already deployed sensing techniques (i.e., indoor environment sensors, PIR, WIFI, Bluetooth, Smart Meters, etc.). Different studies have shown the importance of these technologies for collecting and gathering accurate indoor occupancy information. For example, the authors in [10] showed used a combination of different types of sensors and ANNs in order to predict and estimate the number of occupants in an office room, achieving a high mean coefficient of determination (R2) of 83%. In fact, the associated Wi-Fi devices proved to be efficient at estimating the number of indoor occupants. Moreover, the KNN algorithm and data from multiple environmental sensors are presented and evaluated in the work presented in [11] to predict the number of building occupants. The results show the effectiveness of this approach, achieving an accuracy between 95.4% and 97.5% in the detection of occupants’ presence while an RMSE between 0.121 and 0.79 was estimated for the number of occupants. The author of [12] evaluated the use of clustering algorithms and collected electricity consumption data from a smart meter. The results showed that the accuracy varied between 69% and 90%. Furthermore, the hidden Markov model (HMM) and statistical regression methods are proposed in [13] to estimate the number of occupants in different conference rooms using deployed PIR sensors. The results showed the usefulness of using this approach, with a mean absolute error (MAE) of 0.64–0.99.

The integration of modern information and communication technologies, such as IoT and big data technologies, into building management systems can help to improve the performance and efficiency of buildings’ services (e.g., lighting, ventilation) [14]. Wireless sensor networks, image processing, object tracking, target detection, and identification are some techniques that can be integrated into BMSs. In fact, combining multiple data sources is a leading approach to obtaining improved information (i.e., less expensive, higher quality, or more relevant information) [15]. The development of many data processing and IoT technologies has created a new development opportunity for data fusion. Furthermore, several problems related to energy management can be solved using data fusion methods, such as occupancy prediction, load forecast, predictive control, and occupants’ comfort scenarios [16,17,18]. In fact, most machine learning algorithms that have recently emerged for data fusion and prediction intend improve the performance of different services, such as healthcare, urban road planning, and energy management systems [19,20,21]. Similarly, the management of data collected from various sources represents a significant challenge. Distributed systems, such as Hadoop and MapReduce, have been proposed to manage and process data using power parallel computing techniques. However, handling real-time big data processing, which requires a large stream of information for continuous processing, is still a challenging task. Novel architectures are required to process various data streams. The above-mentioned technological progress has allowed the development of numerous IoT-based platforms, which have emerged for easy deployment of context-driven control applications in smart buildings. In fact, several scenarios related to building management systems, even in research practices or industrial projects, require the integration of IoT technologies and machine learning algorithms to anticipate the changes and predict the actions that better fit users’ needs. However, classical batch setting of machine learning requires a large amount of data, which requires an increased processing time. In fact, with the objective of enabling the development of applications and services that require real-time or near real-time data processing, several algorithms have been developed that target the processing of stream and non-stationary environmental data [22]. In real-world applications, since data tends to change over time, the prediction results of models trained in the past may become less efficient in terms of accuracy. The challenging task in this type of application is the problem of concept drift, especially when the relationships between input and output data in the underlying problem change over time [23,24,25].

**Figure 1:**
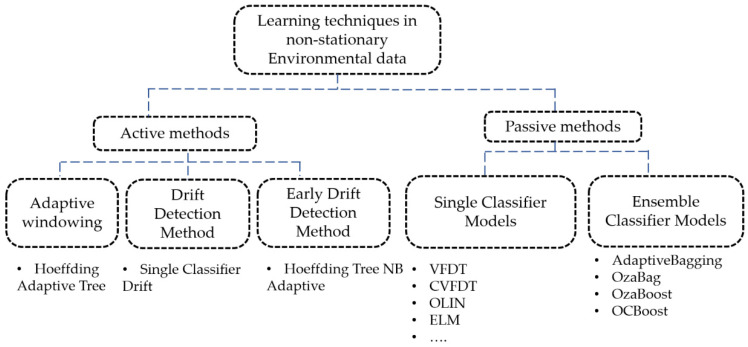
Learning techniques in non-stationary environmental data.

As presented in Figure 1, learning in non-stationary settings involves adaptive approaches that adjust the model accordingly to fit the fundamental changes [26]. In fact, learning algorithms in the presence of drift change are mainly based on either active or passive approaches [27]. Active methods in machine learning require the user to label data with the desired output interactively. However, passive approaches are usually focused on detecting the time of change while algorithms are used to update the model continuously. Moreover, active machine learning methods in the presence of concept drift are mainly based on change detection approaches that initiate, whenever recommended, an adaptation method, which pursues a reaction when the change is detected by modifying the model or creating a new classifier. Generally, the adaption phase can be activated only when a change is detected. The classifier deletes the obsolete knowledge and adapts to the new environment accordingly. To fit the ambiguity in the change detection, passive approaches continuously adapt model parameters whenever new data arrives. In fact, continuously maintaining an up-to-date model in passive approaches avoids the drawbacks associated with active approaches (i.e., falsely detecting a non-existent change, failing to detect a change). Two focal categories can be defined in passive approaches: those that add, remove, or modify members of an ensemble-based system; and those based on updating a single classifier [23]. Furthermore, the human learning philosophy is based on accumulating descriptions from facts and incrementally enhancing those descriptions when new facts and observations become available [28]. In fact, incremental learning represents a big resolver method for learning from sequential flow information and dealing with limited memory and power processing. The principal ability of incremental learning is that it can learn and update the model with new data, which is labeled or unlabeled. Moreover, unsupervised incremental learning or incremental clustering is based on cluster algorithms that generate a new decision in response to new patterns, which are sufficiently different from the previously seen instances, while supervised incremental learning is a learning method for when new instance data is labeled (i.e., the output is known for all inputs). Often, models are used after one-off training. In fact, to maintain the accuracy of prediction results over time, the data used for making the prediction should have a similar distribution to the data used for model training. However, dealing with the new behavior of an environment mostly requires an adaptive method [29]. Recent research works regarding stream machine learning have investigated the drift handling issue, which is based on the assumption of the probably approximately correct (PAC) learning model. Moreover, the error rate of the learner can decrease with the increase in the number of samples when the distribution of the samples is stationary. In fact, this method detects the increase in the error rate, which exceeds the computed threshold. An alert can be generated when a concept drift is detected, and a warning zone detected if the error rate occurs in the future.

This work is part of a project that we designed to develop a holistic platform for collecting, visualizing, and analyzing data from deployed sensors to control active systems in smart buildings (e.g., the HVAC system). In fact, machine learning algorithms have been presented in several research studies to detect and estimate the number of occupants in a building. For example, the authors in [30] showed that the use of the artificial neural network (ANN) algorithm can help to predict the number of occupants, with an accuracy of 70%. Furthermore, the work presented in [31] shows that the occupancy detection accuracy when using HMM and different environmental sensing data varies between 19.1% and 92.54%. The authors in [32] evaluated the performance of four occupancy detection algorithms: gradient boosting machines (GBMs), random forest (RF), linear discriminant analysis (LDA), and classification and regression trees (CARTs). Environmental data collected from an office was exploited to train the models. The results showed an accuracy of 32.68–99.33%. Indeed, various research works have used batch machine learning algorithms, which aim to first store the collected data and then use this data to train the model. Later, the model can be used to predict the occupancy without access to updates with new arrived instances for the model. It is difficult to deal with non-stationary environments using such methods. Therefore, the prime purpose of this work was to implement and deploy a platform by integrating IoT technologies with stream machine learning algorithms, which are appropriate for non-stationary environmental data, for direct application to generated data from different sources (e.g., sensors, RFID, etc.). Three machine learning algorithms for stream data processing are presented, tested, and evaluated in term of their accuracy and resource performance (i.e., RAM, CPU), with the aim of predicting the number of occupants in smart buildings. In short, the key added value of this work is two-fold:

- Evaluating the stream machine learning algorithms in terms of the accuracy and performance, with the aim of predicting number of occupants in smart buildings.
- Deploying and introducing the platform architecture adopted for the application of stream machine learning algorithms to predict the number of occupants.

## 2. Related Work

Occupancy information in smart buildings is a crucial parameter that can be used for controlling HVAC and lighting systems (HVAC-Ls). In fact, using occupancy as a driver for controlling HVAC-L systems has been explored in many research works. However, due to the difficulty in obtaining and predicting accurate real-time occupancy data, many occupancy-driven control techniques focus on using pre-determined schedules [33]. For instance, the number of occupants can be used to more accurately control active systems, with the aim of optimizing the energy use in buildings [34]. In many modern buildings, several solutions use occupancy information for lighting systems’ control. Occupancy (e.g., presence) information can be obtained by using, for example, passive infrared sensors (PIRs), which are connected directly to local lighting fixtures. However, this information is rarely used for intelligent HVAC management [33]. Several sensing technologies have recently emerged and can be integrated to extract a variety of environmental and contextual data. Examples of sensors that can be used to collect and monitor a valuable building’s information to detect occupancy include temperature, humidity, CO_2_, motion, and current sensors [35]. Many recent studies have revealed the effectiveness of combining wireless sensing and IoT tools with a diversity of existing machine learning methods (e.g., classification algorithms) to improve the occupancy detection accuracy by developing and integrating data-driven occupancy prediction into BMSs [4]. For example, the authors in [36] collected electricity consumption datasets using smart electricity meters deployed in five households to train a classification model based on K-nearest neighbor (KNN), support vector machines (SVMs), hidden Markov model (HMM), and thresholding (THR) to detect the presence of occupants. In fact, the accuracy of the occupancy prediction was higher than 80% compared to the accurate value, which was gathered from a tablet computer deployed in the main entrance. Different statistical classification models are presented in [37], mainly LDA (linear discriminant analysis), RF (random forest), and CARTs (classification and regression trees). A dataset with four attributes (i.e., light, temperature, humidity, and CO_2_) was used for training and prediction purposes. It was found that the time of day and status of the week (weekend, weekdays) increased the accuracy of occupancy detection by 32%, reaching 97% with the use of 2 predictors. A digital camera was used for gathering the ground truth occupancy value to train a classification model. Most of these machine learning methods have used batch setting approaches (i.e., data should be available in the database to be processed). However, as stated above, several algorithms were developed to be applied in a non-stationary environment, especially when the context changes frequently. These algorithms are mainly based on either passive or active methods. Algorithms based on active approaches aim to detect concept drift while algorithms based on passive methods update the model continuously whenever new data are presented regardless of whether there is a drift. In contrast, algorithms based on active approaches aim to detect concept drift [23]. Recent studies have indicated the effectiveness of moving from traditional machine learning methods to learning methods, which are principally applied for stream data processing. In general, the development of methods that maintain an accurate decision model with the ability to learn and forget concepts incrementally is a crucial challenge when dealing with real-time data stream processing [24,33]. Indeed, several research works, which are related to machine learning for stream data processing, are based on supervised learning [25], mainly for classification purposes. Most of these works focused on addressing the problem of changes in the implicit data distribution over time (i.e., concept drifts) [27]. However, time-series prediction requires the development of stream machine learning. For instance, to estimate the occupancy of a building, data from multiple sensors, such as temperature, humidity, CO_2_, current, lighting, and sound sensors, is required [38,39]. Several works have shown the effectiveness of using univariate methods to detect occupancy. For example, the authors in [40] shed more light on the occupancy detection problem (presence) by adopting well-known machine learning techniques. Similarly, in [41], the authors showed the usefulness of using the concentration of carbon dioxide (i.e., indoor air quality). Residential and non-residential buildings were used as a testbed to validate the proposed algorithm [37]. The obtained results show its performance in detecting the presence and number of occupants was 95.8% and 80.6%, respectively. The study presented in this article is part of a project we are conducting that is focused on the development of an intelligent building management platform that integrates IoT and stream data processing techniques, with the aim of reducing energy consumption while maintaining occupants’ comfort. In fact, combining IoT and big data technologies with real-time machine learning is considered key to the analysis and processing of events’ streams and the prediction of actual demands. We focus foremostly on the platform we have developed and deployed for predicting the number of occupants.

## 3. Methods and Materials

### 3.1. Platform Architecture for Collecting and Processing Data

**Figure 2:**
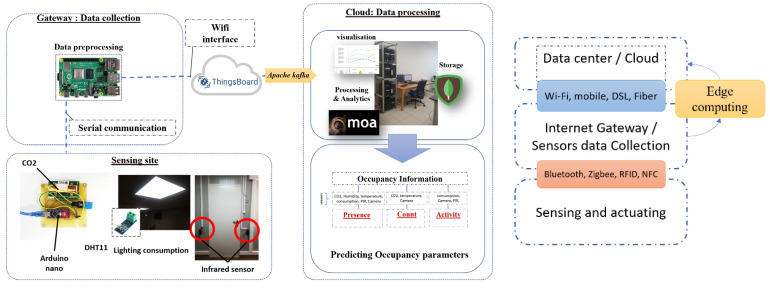
The proposed platform architecture.

In this section, we introduce the platform prototype used to gather environmental data to assess the usefulness of predicting the number of occupants. Figure 2 presents the platform architecture, which is composed of 3 main layers, namely, the perception, processing, and application layers. In fact, the first layer, named the physical layer, includes different sensors that gather indoor/outdoor environmental data, and actuators for controlling the building’s services. This layer includes a preprocessing unit for gathering and transforming raw data into an understandable format, and a real-time processing unit for processing these data streams using recent stream processing technology. Many types of sensors are used in the experimental setup to collect environmental data: DHT11 to obtain temperature and humidity measurements, an MH-Z14A NDIR sensor to obtain the indoor CO_2_ level, current sensor to generate the power consumption, and infrared sensors to obtain accurate data regarding the number of occupants, which is used for accuracy comparison purposes. Furthermore, several stream processing tools can be used and adapted for occupancy prediction. The latter can be exploited as inputs for controlling active equipment, such as HVACs, shading systems, and lighting. These data are used to assess a significant context, such as estimation of the number of occupants and indoor thermal conditions, to develop a context-driven control approach to active equipment (e.g., ventilators, HVAC, lighting).

**Figure 3:**
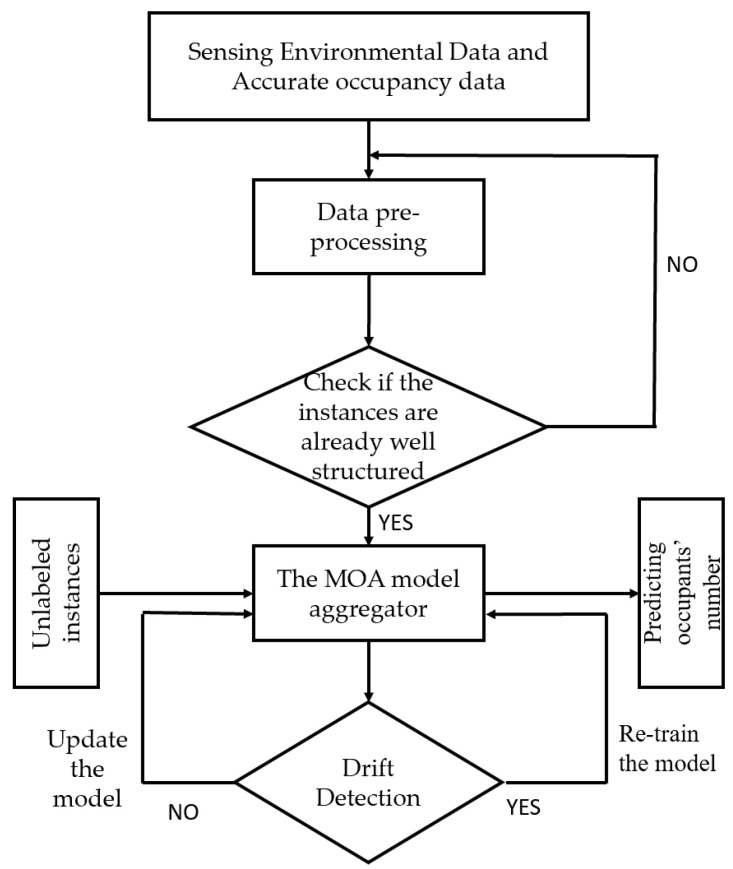
The global flowchart of the proposed methodology.

In this platform, Thingsboard (www.thingsboard.io, accessed on 12 December 2021) API is used and deployed in a raspberry Pi 3, which collects data from various sensors and sends it to the server side. Stream machine learning algorithms are integrated into the platform to execute the real-time processing algorithms to predict the number of occupants. A scenario was deployed in our test site (EEBLab, Energy Efficient Building Lab) for real sitting experiments, as shown in Figure 2. Moreover, the flowchart presented in Figure 3 describes as generally as possible the approach implemented to predict the number of occupants.

### 3.2. Predictive Modeling Methods

This section is dedicated to a brief presentation of the machine learning algorithms we used for occupancy prediction. Generally, a predictive modeling problem is described as follows: let us assume that we have a set of N training examples in the form of (x,y), where a model “y = f(x)” can be produced via the training phase. This model can be used for predicting class “y” of future examples x with high accuracy. The approach presented in this work aims to predict the number of occupants using four features (i.e., CO_2_, temperature, humidity, and light consumption) extracted from environmental sensors deployed in a simulated office room in our laboratory. Moreover, 3 classifiers (i.e., Hoeffding tree, naïve Bayes, and SAMKNN) are integrated and evaluated using separate classes of occupancy (e.g., 1 occupant, 2 occupants, 3 occupants…8 occupants). In fact, these classifiers are implemented by the MOA community and designed for stream machine learning.

#### 3.2.1. Hoeffding Tree Algorithm

A classic decision-tree-based classifier (e.g., C4.5, CART, and ID) assumes that the data used for training is already stored in a particular memory and is severely limited in the number of examples. Furthermore, disk-based decision tree techniques, such as SLIQ and SPRINT, assume that the examples are stored on the disk and are learned by repeatedly reading them in sequential manner. Training sets can become expensive when training complex trees, which greatly increases their size [42]. In fact, the main purpose is to develop a decision tree for a large amount of data. The learner should analyze the data efficiently and as fast as possible.

**Figure 4:**
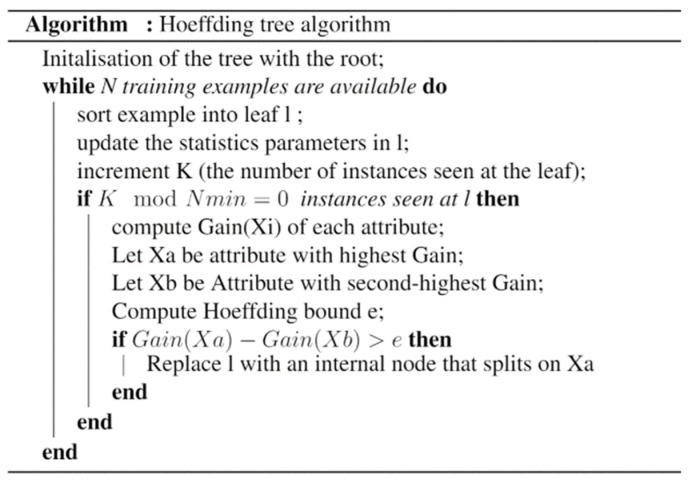
The pseudo code of the Hoeffding tree algorithm [43].

The Hoeffding tree can learn from a significant data stream when the distribution of the generating examples does not change over time. Furthermore, the Hoeffding tree is based on the Hoeffding bound measurement to efficiently construct a decision tree with the minimal number of instances needed to achieve a certain level of confidence. In fact, as shown in Figure 4, to find the best attribute to test on a given node, it may be sufficient to consider only a small subset of the training examples that pass through that node using Hoeffding bound measurement. Thus, given a stream of examples, the first ones will be utilized for choosing the root test, then the succeeding examples will be passed down to the corresponding leaves and used to select the appropriate attributes there, and so on recursively.

#### 3.2.2. Naïve Bayes Algorithm

**Figure 5:**
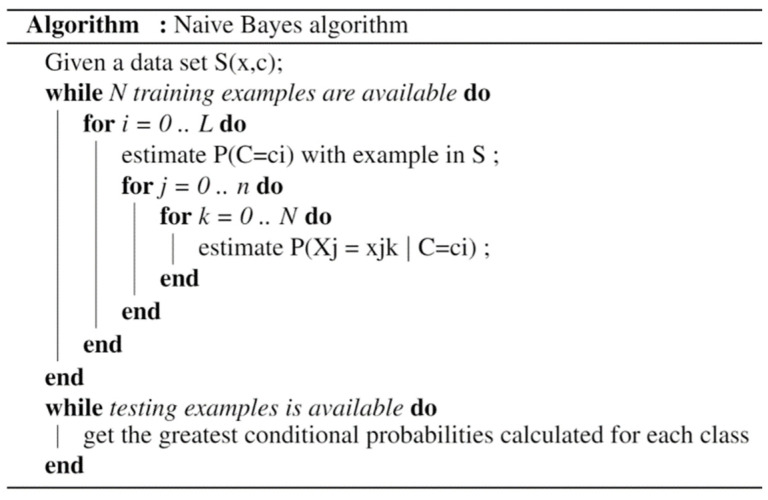
The pseudo code of the naïve Bayes algorithm [45].

Furthermore, naïve Bayes classifiers are a family of simple probabilistic classifiers, which are based on the use of the Bayes theorem (1). The principal idea is to calculate a probability for each one of the classes based on the attribute values, along with selecting the class that has the highest probability. As shown in Figure 5, with the naïve independence assumptions between the features, the class probabilities can be calculated by multiplying over all attributes the probability of having that particular class label conditioned on the attribute having a particular value:

(1) $$P\left(c|x\right)=\frac{P\left(x|c\right)P\left(c\right)}{P\left(x\right)}$$

where *P*(*c*|*x*) is the posterior probability of the target class given the predictor attributes, *P*(*c*) is the prior probability of the class, *P*(*x*|*c*) is the likelihood, and *P*(*c*) is the prior probability of attributes. In fact, when all probabilities are computed, the algorithm chooses the class with the highest probability as the predicted class.

The added value to deal with real-time stream data processing is described in [44,45]. The authors propose a novel method that develops naïve Bayes classifiers based on count min sketch (CMS) to minimize the required space to store training data. Further, the proposed algorithm adapts concept drift approaches to deal with the fact that streaming data changes over time.

#### 3.2.3. KNN Classifier with Self-Adjusting Memory (SAMKNN) Algorithm

KNN is a lazy learning algorithm, with the concept of feature similarity (e.g., similar things are near each other). It stores the entire training dataset, which it uses as its representation, and makes predictions just-in-time by calculating the similarity between an input sample and each training instance. In fact, the combination of the self-adjusting memory (SAM) and k-nearest neighbor (kNN) classifier can deal with concept drift [46]. It presents several analogies to the human memory structure since the knowledge is divided between short- and long-term memory. In fact, SAM-kNN can deal with varied concept drifts using the coordination of biologically inspired memory models.

**Figure 6:**
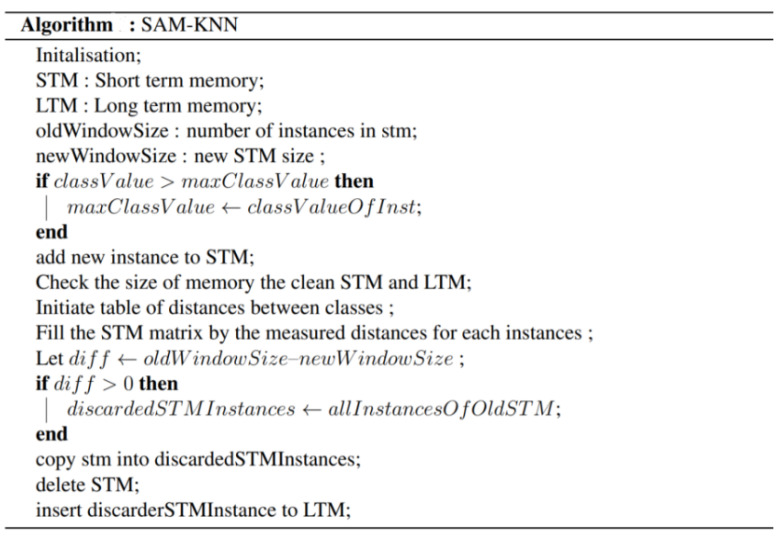
The pseudo code of SAMKNN [46].

As depicted in Figure 6, the SAM architecture is represented as follows: the SAM architecture is based on 4 main phases: model adaptation, which aims to manage every type of memory with the corresponding weight. For example, the role of model adaptation of the short-term memory (STM) is to completely contain the data of the current concept. Therefore, its size must be reduced whenever the concept changes, such that examples of the former concept are erased. The transfer and cleaning phase includes the data of former concepts that is reliable with the STM in the LTM, and cleaning of the LTM is required according to every seen instance. Furthermore, as the STM is reduced in size, the sorted-out data cannot simply be discarded, as it may contain valuable information for future prediction. Instead, we can transfer as much information as possible to the LTM. In the compression phase, available information is condensed to a scattered knowledge representation via clustering because instances, in the STM principle, do not disappear as soon as the size limit of the LTM is reached (unlike the FIFO). In fact, in the model’s weight adaptation, the weight of a memory is represented by the accuracy averaged over the last samples of the current STM.

## 4. Experimental Results and Discussion

As stated above, different research studies related to the issue of occupancy predicting in smart buildings have used batch setting of machine learning algorithms, which first targets the storage of the data and then uses a significant amount of stored data to train the model. It is difficult to cope with non-stationary environments using such methods. Therefore, the purpose of this study was to evaluate and explore the effectiveness of stream machine learning, which is appropriate for non-stationary environmental data, to predict the number of occupants in smart buildings.

The experiments were conducted all day long (i.e., from 8:00 to 20:00) with a sampling rate of 1 s, and validated using diverse sensors’ data, such as CO_2_, temperature, humidity, and light consumption. The accurate value of the number of occupants was used to better analyze the correlation between the number of occupants and other environmental parameters. The machine online analysis (MOA) framework was deployed for data stream processing.

In these experiments, the number of occupants was considered as a class label. In fact, the 3 abovementioned algorithms, which have already been implemented as classifiers in MOA, were tested and compared (i.e., Hoeffding tree, naïve Bayes, and SAM-KNN) using the test and train or prequential task and evaluating the frequency sampling of 500 instances. The purpose of this study was assess their effectiveness in predicting the number of occupants.

It is worth noting that the experiments were conducted on a day when our EEBLAB was occupied. The number of occupants varied between zero and two in the first half of the day. In the afternoon, the number of occupants reached seven occupants. Therefore, this represented a good period for conducting an experiment to predict the number of occupants. We compared the three abovementioned algorithms.

**Figure 7:**
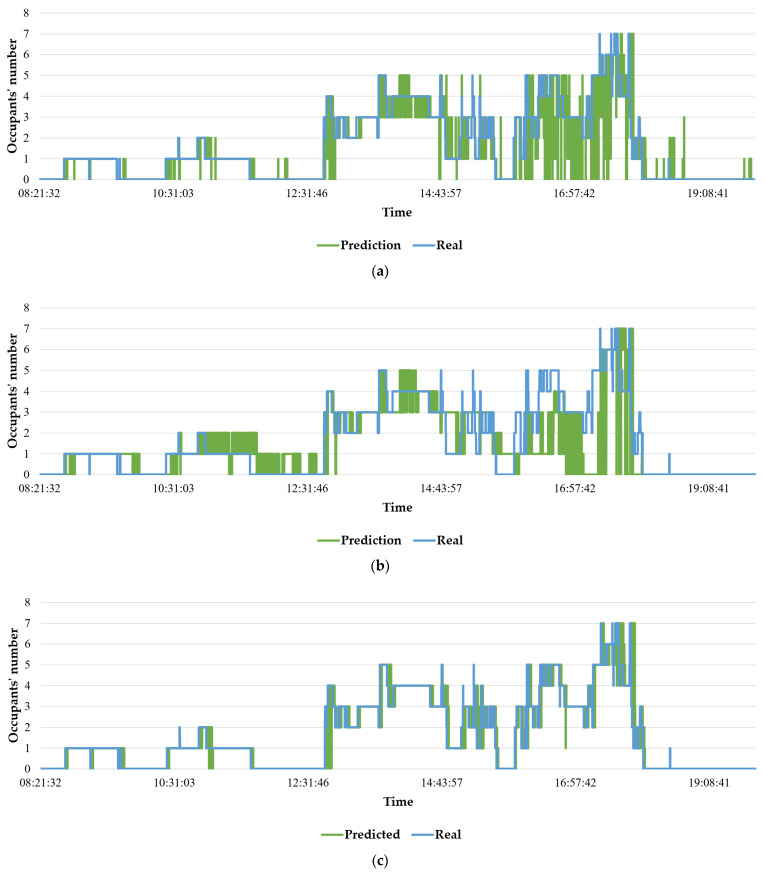
Occupancy prediction using: (**a**) Hoeffding tree, (**b**) naïve Bayes, and (**c**) SAMKNN.

**Figure 8:**
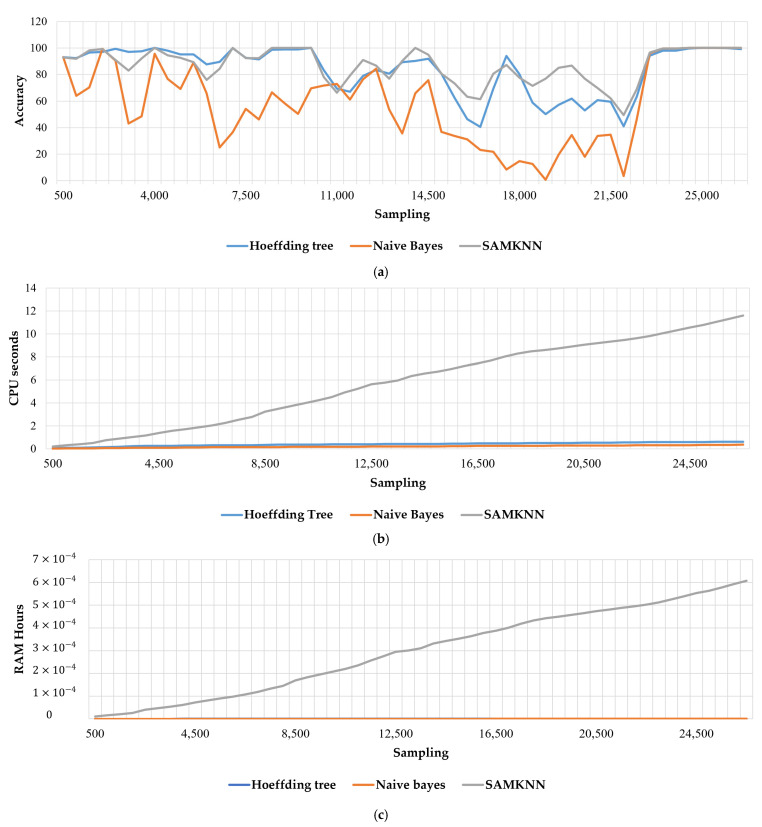
Evaluation results of the presented algorithms: (**a**) accuracy, (**b**) CPU seconds, (**c**) RAM hours.

Figure 7 shows that the predicted values using the Hoeffding tree and naïve Bayes algorithms are not truly accurate, as a significant gap can be observed between the predicted and accurate value. However, the predicted values, which were generated using SAM-KNN algorithm, are almost close to the true values of number of occupants. Figure 8 shows the effectiveness, in terms of the accuracy, of the three algorithms in predicting the number of occupants.

Generally, most of the current research in terms of occupancy prediction has used accuracy as one performance index, which represents the percentage of the correct value, returned by the predictor returns. It is defined by Equation (2):

(2) $$Accuracy=\frac{1}{N}\sum_{k=1}^{\left|G\right|}\sum_{x:g\left(x\right)=k}I\left(g\left(x\right)=g^{\prime \prime }\left(x\right)\right)$$

where “*I*” is an indicator function, which equals 1 when the classes match and 0 otherwise.

Machine learning is very computationally intensive. It is worth noting that a fast CPU is needed to train and evaluate the model. In our experiments, we used a suitable machine (i.e., Core i7 7700 HQ and 16 GB of RAM) to test and assess the effectiveness of the considered algorithms.

In fact, for the Hoeffding tree algorithm, the accuracy increased to above 98% (with an average of 83.74 %) in predicting the number of occupants after 23,500 instances using the prequential task. Moreover, 39 nodes and 20 leaves were created by the model after 26,000 instances, with 0.04 CPU s and $3.85\cdot 10^{-11}$ GB of RAM per hour used for training and evaluating the model for 500 instances. Furthermore, the naïve Bayes algorithm utilized $5.48\cdot 10^{-12}$ GB of RAM per hour for testing and training the model and $6.65\cdot 10^{-2}$ s of CPU for 500 instances. The accuracy decreased to under 50% (with an average of 58.85 %) in many periods. Indeed, KNN with the self-adjusting memory architecture showed its performance in terms of accuracy. It varied above 50%, with an average of 87.06%, as illustrated in Figure 8a and Table 1. However, it consumed more than the Hoeffding tree and naïve Bayes algorithms in terms of memory use (RAM hours) and CPU use (seconds) as shown in Figure 8 and Table 1. SAMKNN consumed $1.12\cdot 10^{-5}$ GB of RAM per hour for testing and training of the model and around 0.21 s for CPU use time.

## 5. Conclusions and Perspectives

In this work, a novel platform architecture integrating an IoT platform (i.e., Thingsboard) to collect sensors’ stream data and machine learning algorithms implemented in the server site for application to stream and non-stationary data was introduced. Further, these algorithms were deployed, and used to predict the number of occupants in an office room. In fact, three algorithms (i.e., Hoeffding tree, naïve Bayes, and SAM KNN) were evaluated and compared in terms of the accuracy, model cost (i.e., how much GB can be used per hour), and execution time (i.e., CPU use time). The experimental results indicate the usefulness of the proposed algorithms compared to algorithms that only adopt batch setting. Thereby, the number of occupants was predicted with an accuracy of more than 83% and without resource wasting. The CPU use time was 0.04 s and $3.85\cdot 10^{-11}\;$GB of RAM could be exploited per hour using a suitable machine (i.e., Core i7 7700 HQ and 16 GB of RAM). Further, occupancy prediction can help to control active systems, such as HVAC and lighting systems. In fact, most control algorithms (e.g., PID, MPC, GPC) use the number of occupants as the main input to control these buildings’ equipment. The integration of IoT and big data technologies in such scenarios in smart building management systems can help to improve the development of new approaches to controlling active systems in smart buildings. In our ongoing work, further experiments will be conducted to present the usefulness of online machine learning in developing context-driven approaches to controlling shading, lighting, and HVAC control to reduce energy consumption while maintaining occupants’ comfort.

## Figures and Tables

**Table 1:** Average results of the algorithms’ evaluation.

Algorithms	CPU Seconds	RAM-Hours	Accuracy Rate
Hoeffding tree	0.04 s	$3.85\cdot 10^{-11}$ GB	83.74 %
Naïve Bayes	$6.65\cdot 10^{-2}$ s	$5.48\cdot 10^{-12}$ GB	58.85 %
SAMKNN	0.21 s	$1.12\cdot 10^{-5}$ GB	87.06 %
